# Systematic Assessment of Stellate Ganglion Block in Post-Traumatic Stress Disorder: Exploring Clinical Utilization and Significance

**DOI:** 10.1192/j.eurpsy.2025.1962

**Published:** 2025-08-26

**Authors:** S. Prasad, N. Jain, G. S. Gill, A. K. Bachu, S. Gunturu

**Affiliations:** 1 Psychiatry, BronxCare Health System, New York, United States; 2 Riga Stradins University, Riga, Latvia; 3 Case Western Reserve University, Cleveland; 4 Psychiatry, University of Arkansas for Medical Sciences, Little Rock, United States

## Abstract

**Introduction:**

Post-traumatic stress disorder (PTSD) is a chronic condition resulting from exposure to traumatic events. The utilization of stellate ganglion block (SGB) as a potential treatment for PTSD has garnered increased interest in recent years. SGB acts by blocking sympathetic outflow, offering promise in alleviating autonomic dysfunction associated with PTSD symptoms. However, the evidence supporting SGB’s efficacy compared to established recommendations remains limited.

**Objectives:**

To bridge this knowledge gap, a systematic review was conducted following PRISMA guidelines to assess the clinical applications and implications of stellate ganglion block (SGB) in the management of post-traumatic stress disorder (PTSD). The study aimed to identify pertinent literature, synthesize findings from diverse sources, evaluate outcomes of SGB therapy for PTSD, analyze factors such as anesthesia preferences and procedural methods, scrutinize symptom alleviation post-SGB sessions, explore reported side effects and symptom recurrence, and shed light on existing limitations within the current discourse on SGB’s utility in treating PTSD.

**Methods:**

The systematic review involved the evaluation of 14 studies meeting predetermined inclusion criteria, incorporating a total of 550 participants. Notably, the majority of participants were military service members and veterans, with a median age of 36.9 years. The review focused on anesthetic practices, procedural techniques, timing of SGB administration, and symptom progression post-SGB therapy sessions.

**Results:**

Analysis of the selected studies highlighted the prevalent use of 0.5% ropivacaine as the preferred anesthetic for SGB, with the right-sided technique being the most commonly employed. Timing of the initial SGB session varied widely, with symptom improvement typically observed immediately or within the first week post-procedure. Positive outcomes often coincided with reductions in alcohol intake, medication use, and enhanced mood. Recurrence of symptoms was noted, necessitating additional SGB sessions, while reported side effects were predominantly mild and transient in nature.

**Conclusions:**

While promising, caution is advised when interpreting the benefits of SGB due to challenges such as the absence of standardized clinical trial data, variabilities in reported outcomes, and potential reporting biases. Addressing these limitations through standardized assessment and reporting in future studies is crucial to enhance comprehension of SGB’s efficacy, safety, tolerability, and appropriate indications for treating PTSD. This endeavor is pivotal in advancing a more nuanced understanding of SGB’s role as a therapeutic modality in PTSD management.

**Disclosure of Interest:**

None Declared

